# A Convolutional Neural Network Deep Learning Model Trained on CD Ulcers Images Accurately Identifies NSAID Ulcers

**DOI:** 10.3389/fmed.2021.656493

**Published:** 2021-08-27

**Authors:** Eyal Klang, Uri Kopylov, Brynjulf Mortensen, Anders Damholt, Shelly Soffer, Yiftach Barash, Eli Konen, Ana Grinman, Reuma Margalit Yehuda, Martin Buckley, Fergus Shanahan, Rami Eliakim, Shomron Ben-Horin

**Affiliations:** ^1^Department of Diagnostic Imaging, Sheba Medical Center, Tel Hashomer, Affiliated to Sackler Medical School, Tel Aviv University, Tel Aviv, Israel; ^2^Deep Vision Lab, Sheba Medical Center, Tel Hashomer, Israel; ^3^Department of Gastroenterology, Sheba Medical Center, Tel Hashomer, Affiliated to Sackler Medical School, Tel Aviv University, Tel Aviv, Israel; ^4^Chr. Hansen A/S, Human Health Innovation, Hoersholm, Denmark; ^5^APC Microbiome Ireland, Cork, Ireland; ^6^Centre for Gastroenterology, Mercy University Hospital, Cork, Ireland

**Keywords:** deep learning, capsule endoscopy, Crohn disease, anti-inflammatory agents, non-steroidal, NSAID

## Abstract

**Background and Study Aims:** Deep learning (DL) for video capsule endoscopy (VCE) is an emerging research field. It has shown high accuracy for the detection of Crohn's disease (CD) ulcers. Non-steroidal anti-inflammatory drugs (NSAIDS) are commonly used medications. In the small bowel, NSAIDs may cause a variety of gastrointestinal adverse events including NSAID-induced ulcers. These ulcers are the most important differential diagnosis for small bowel ulcers in patients evaluated for suspected CD. We evaluated a DL network that was trained using CD VCE ulcer images and evaluated its performance for NSAID ulcers.

**Patients and Methods:** The network was trained using CD ulcers and normal mucosa from a large image bank created from VCE of diagnosed CD patients. NSAIDs-induced enteropathy images were extracted from the prospective Bifidobacterium breve (BIf95) trial dataset. All images were acquired from studies performed using PillCam SBIII. The area under the receiver operating curve (AUC) was used as a metric. We compared the network's AUC for detecting NSAID ulcers to that of detecting CD ulcers.

**Results:** Overall, the CD training dataset included 17,640 CE images. The NSAIDs testing dataset included 1,605 CE images. The DL network exhibited an AUC of 0.97 (95% CI 0.97–0.98) for identifying images with NSAID mucosal ulcers. The diagnostic accuracy was similar to that obtained for CD related ulcers (AUC 0.94–0.99).

**Conclusions:** A network trained on VCE CD ulcers similarly identified NSAID findings. As deep learning is transforming gastrointestinal endoscopy, this result should be taken into consideration in the future design and analysis of VCE deep learning applications.

## Introduction

Non-steroidal anti-inflammatory drugs (NSAIDS) are among the most commonly used medications in the world, very frequently purchased over the counter. Gastrointestinal adverse events are common with these medications and involve both upper and lower gastrointestinal tract (1). In the small bowel, NSAIDs may cause a variety of mucosal lesions, including mucosal breaks, mucosal edema, diaphragm-like strictures and ulcers that may resemble intestinal inflammatory lesions in patients with Crohn's disease (2). The prevalence of such small bowel abnormalities among NSAIDs users was reported to be as high as 75% in studies that utilized video capsule endoscopy (VCE) for evaluation of the small bowel (3–6).

NSAID-induced enteropathy is the most important differential diagnosis for small bowel ulcers in patients evaluated for suspected Crohn's disease (CD); abstinence from NSAIDs is recommended before performance of VCE, usually for at least 4 weeks (7–9). As NSAID-induced enteropathy is associated with a significant risk of gastrointestinal bleeding (10), several therapeutic agents such as misoprostol and probiotics were evaluated for mucosal protection in NSAIDs users (11, 12).

In recent years, deep learning is becoming frequently used in multiple areas of medicine including radiology, ophthalmology, oncology and gastroenterology (13–16). Convolutional neural network (CNN) techniques demonstrated excellent accuracy for detection of various endoscopic pathologies (16), and we have previously demonstrated their high accuracy for automated identification and classification of VCE-detected small bowel ulcers in CD patients (17, 18). It is expected that DL research for VCE will continue to emerge and will influence clinical practice, ultimately shortening required physicians' time for reading VCE films or possibly abolishing this need altogether. An overview of DL and CNN is presented in the methods section.

NSAIDS-induced enteropathy is commonly encountered in VCE. DL applications for VCE are being rapidly introduced into research and clinics. Most of CNN are trained using images of CD ulcers. However, the ability of DL to identify findings related to NSAID enteropathy have not yet been evaluated.

We evaluated a DL network for the detection of NSAID ulcers on VCE images. Since training a DL network requires a large number of images, and as NSAID enteropathy ulcers are morphologically similar to CD ulcers, we have used a CD ulcers dataset for training the model. Then the machine was solely tested on NSAIDs ulcers dataset that was extracted from a clinical trial in which healthy individuals received NSAID and underwent VCE.

## Patients/Material And Methods

### Overview of Deep Learning Algorithms for Image Analysis

Deep learning (DL), an emerging technology which has gained much interest in the past decade. It is considered a sub-type of artificial intelligence (AI) (13, 14). AI describes algorithms which perform tasks that usually require “human cognitive”.

**Figure 1:**
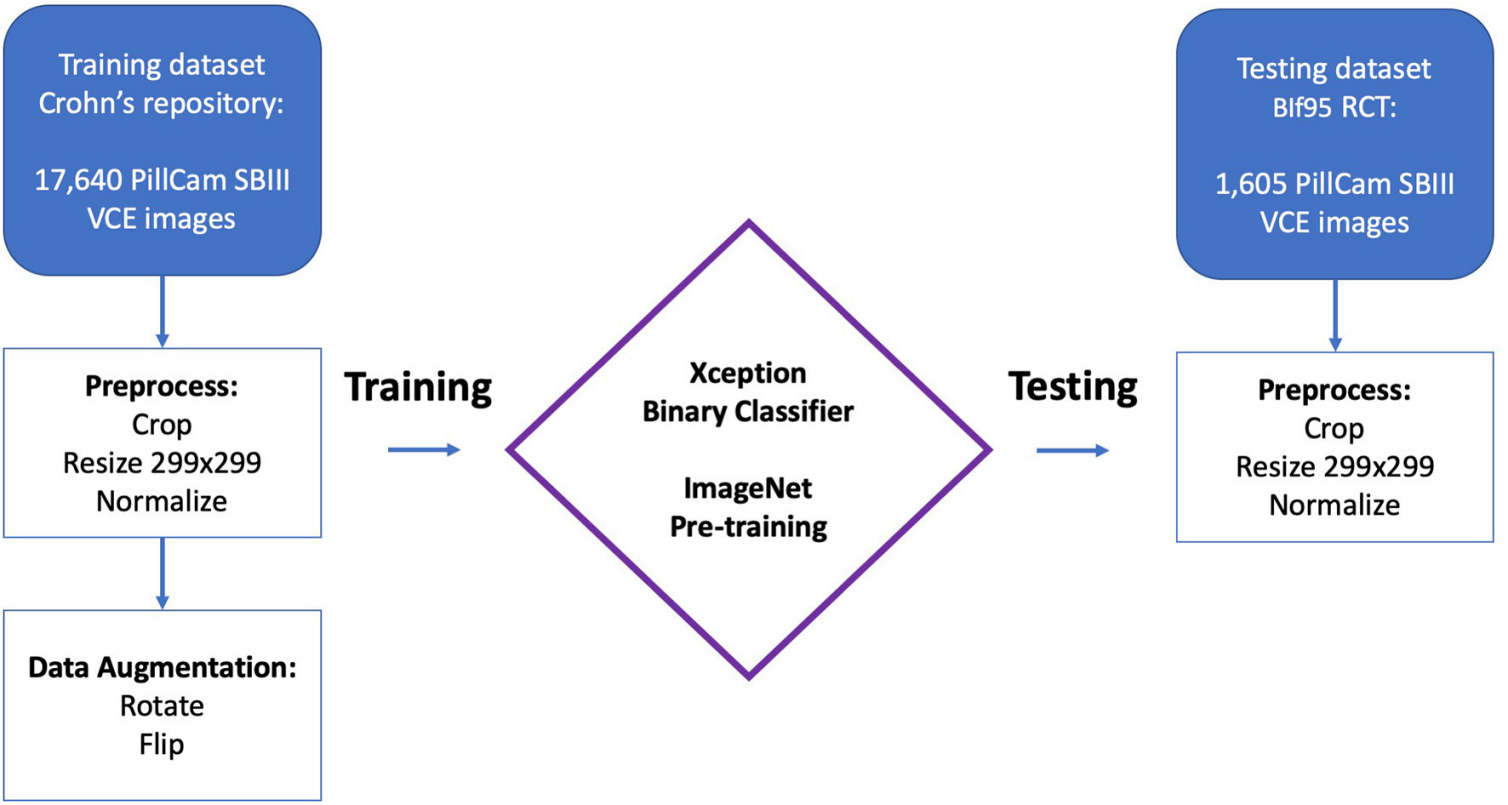
Schematic representation of an artificial neural network (ANN). The network is composed by many linear regression units, termed neurons. These units are structured in sequential layers. Outputs of neurons from the previous layer are the inputs of the neurons in the next layer. The connection between the layers are termed weights, which are similar to the weights of a linear regression unit. Data, such as an image, is propagated through the network, and a final prediction is outputted. An optimized network will produce a desired prediction for each data entry.

DL is almost synonymous with artificial neural networks (ANNs). The structure of ANNs may be considered as a cluster of interconnected linear regression units. This cluster of regression units are joined using non-linear activation functions to create sequential neural layers. Similar to linear regression, each neuron in the network has multiple inputs which are termed weights. These inputs are the outputs of neurons in the previous layer (Figure 1). ANNs may contain many “hidden”/“deep” layers, hence the term “deep learning”.

The process of training and ANN is essentially tweaking of all the network's weights to achieve an optimized network. Given data, an optimized network will produce a desired prediction. For instance, the network can be given VCE images with either ulcers or normal mucosa. An optimized network could satisfactory differentiate between these two types of images.

Convolutional neural networks (CNN) are a sub-type of ANN designed for image analysis. These algorithms are a very similar to classic ANNs. CNNs were specifically built to recognize repeating patterns in data. Since images contain many repeated patterns, CNNs are optimal for this task.

**Figure 2:**
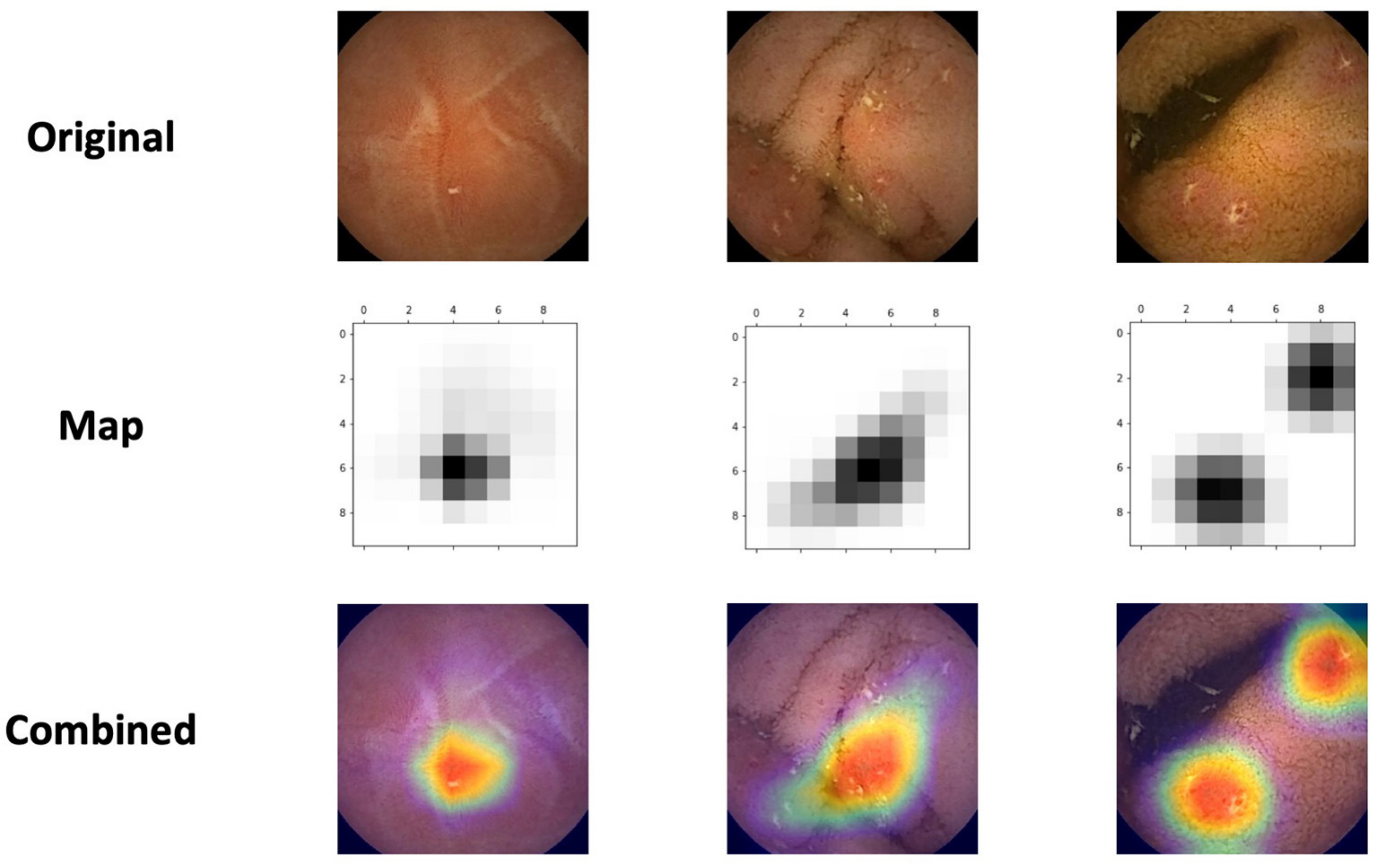
Convolutional neural networks (CNN) are a sub-type of artificial neural networks (ANN). CNNs, are unique in their capacity to identify repeating patterns. Thus, they are ideal for image analysis, as images contain many repeating patterns. CNNs are composed from many small matrixes which are a collection of weights. These small matrices are shared across the array of pixels which constitutes an image.

The CNN is inputted with an image (for example a VCE image), which is an array of pixels. The array is propagated along the deep layers of the CNN until a final neuron outputs a classification prediction (i.e., “ulcer”/“no ulcer”). Each CNN neuron corresponds to a small matrix (i.e., 3X3, 5X5) of weights. The uniqueness of CNN is that these small matrixes are propagated along the image. Thus, each matrix is “shared” across all the regions of the image. This matrix sharing is what allows the pattern recognition of CNNs (Figure 2).

In the past few years, CNNs are being rapidly introduced into healthcare. Since these algorithms are specifically designed for image analysis, the major involved fields are those dealing with medical images, such as radiology images, endoscopy images, fundoscopy, ECG, etc.

### Study Design

We previously published results of a CNN trained on VCE images of CD patients (17). In the current study, we retrospectively evaluated this CNN for its accuracy in detecting NSAIDs ulcers on VCE images. Thus, the network was solely trained on CD ulcers and solely tested on NSAIDs ulcers.

The network was trained using CD ulcer and normal mucosa images sourced from a repository of VCE diagnosed CD patients as previously described (17). All images were acquired from studies performed using PillCam SBIII (Medtronic Ltd, Dublin, Ireland) and reviewed with Rapid 9 (Medtronic Ltd, Dublin, Ireland) capsule reading software.

NSAIDs induced enteropathy images were extracted from VCEs that were performed during the Bifidobacterium breve (BIf95) randomized controlled trial published by Mortensen at al. (11); the methodology and results of the study are covered in detail elsewhere (11). Briefly, this was a single-site, double-blind, parallel group study that enrolled 75 heathy volunteers given acetylsalicylic acid (ASA) (300 mg) daily for 6 weeks. The participants were randomized to groups given oral capsules of Bif95 or placebo for 6 weeks. Small-intestinal damage was analyzed by serial VCEs for the duration of the study. All VCEs were performed using PillCam SBIII (Medtronic Ltd, Dublin, Ireland) and reviewed with Rapid 9 (Medtronic Ltd, Dublin, Ireland) capsule reading software.

For the purpose of the current study, we only selected the VCEs of patients in the placebo arm, i.e. ASA 300 mg for six weeks with placebo and discarded those who received ASA with Bif95. All studies were reviewed in consensus by VCE experts (UK, AG, RE) and images containing mucosal ulcers were extracted.

### Software and Hardware

The models were written in Python (ver. 3.7) utilizing the open-source Keras (ver. 2.1.5) library and the open-source TensorFlow (ver. 1.5.0) library as backend. Models ran on an Intel i7 CPU and two GeForce GTX 1080ti Graphics Cards.

### CNN Model

As previously described (17), an Xception CNN (19) was trained to classify capsule images into either images of normal mucosa or images with CD mucosal ulcers. The network's weights were initialized using weights from the 1.2 million everyday color images of ImageNet (20).

Preprocessing of capsule images included cropping of images' borders and legends. Images were then resized into a 299 × 299 matrix and pixels were normalized into the range 0–1 by dividing by 255 (17).

The following hyper-parameters were used for training the network: 2 epochs; batch size 8; Adam optimization with a learning rate of 10^−4^. Softmax was used as the output activation function.

### Class Activation Maps

We used class activation maps (CAM) to analyze which parts of a given image led the network's decisions. This method is helpful for understanding the decision process of the CNN. Of particular interest, we wanted to investigate whether the network identifies NSAIDs ulcers as CD ulcers.

For this purpose, we've applied Gradient-weighted Class Activation Mapping (Grad-CAM). This algorithm uses the gradients of the target label, flowing into the final convolutional layer, to highlight the important regions in the image (21).

### Metrics

We assessed the networks performance for detecting images containing NSAIDs mucosal ulcers.

Receiver operating curves (ROC) were plotted for the network's results by varying the operating threshold. The model's metrics included area under the curve (AUC), sensitivity, specificity, positive predictive value (PPV) and negative predictive value (NPV), and F1 score. All metrics were computed for the Youden's index and for fixed specificities of 90, 95 and 99%.

Bootstrapping validations (1,000 bootstrap resamples) were used to calculate 95% confidence intervals (CI) for the metrics.

## Results

### Study Cohort

Overall, the CD training dataset included 17,640 CE images from 49 patients; 7,391 images with mucosal ulcers and 10,249 images of normal mucosa. Out of 10,249 normal images, 3,577 originated from patients with normal CE and 6,672 from patients with CD (17).

The NSAIDs testing dataset included 1,605 CE images (11). This included 980 images of NSAIDs ulcers and 625 of normal mucosa.

### CNN Results

The network's prediction time for the 1,605 images in the testing dataset was 8.9 s. This yields an average of 5.5 ms per single image. For a VCE film with 10,000 images, this amounts to 55 s.

**Figure 3:**
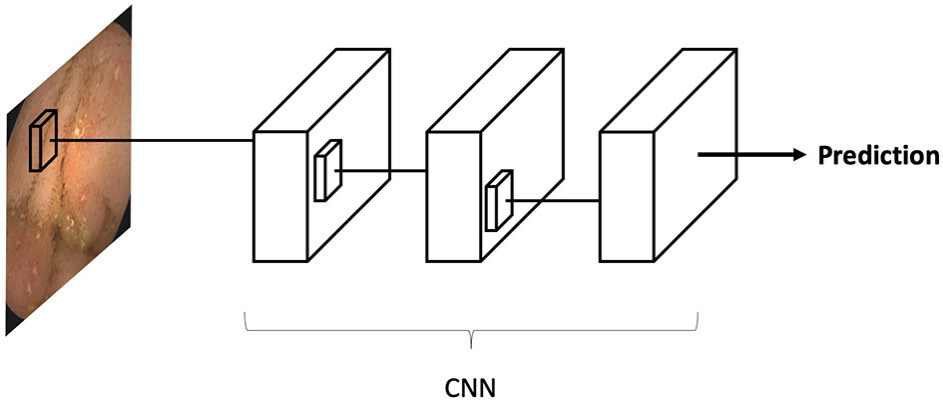
Three examples of the network's activation heatmaps for video capsule endoscopy images of NSAID ulcers.

Figure 3 presents the network's activation heatmaps for NSAID ulcers. As can be seen in the heatmaps, a network trained on CD ulcers, clearly detected NSAID ulcers.

**Figure 4:**
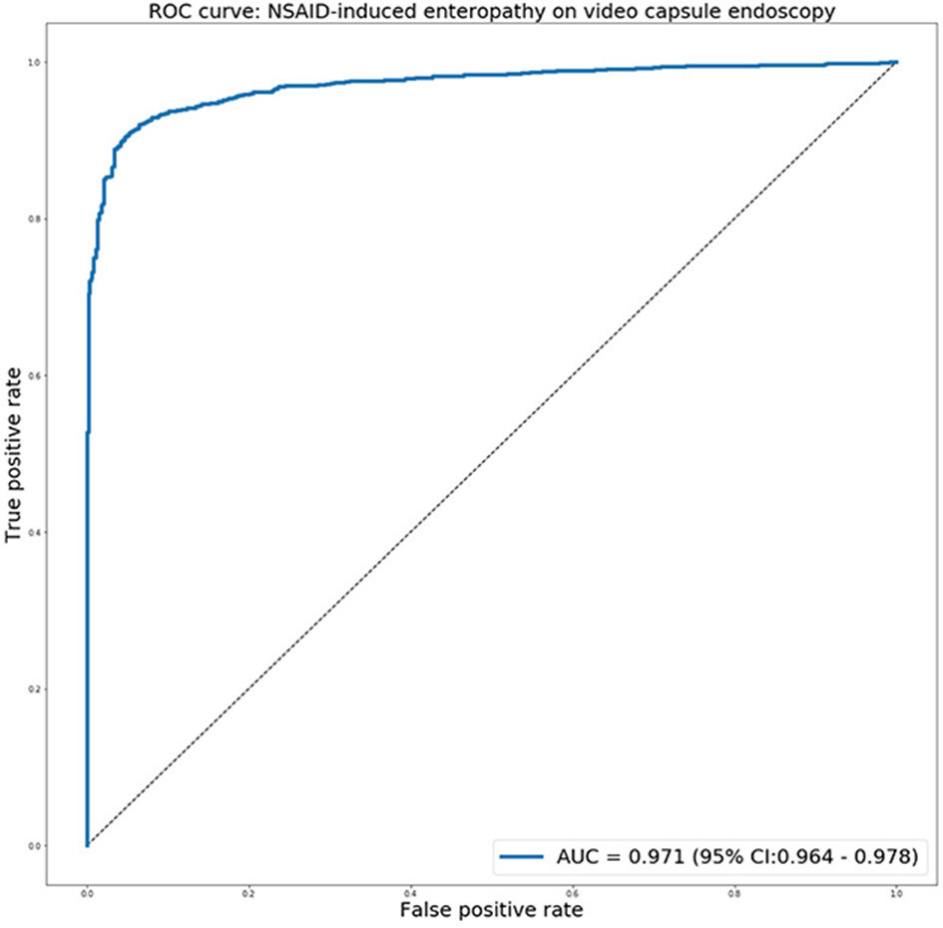
The receiver operating characteristic curve for predicting NSAIDs ulcers using the deep learning network.

The receiver operating characteristic curve for predicting NSAIDs ulcers is presented in Figure 4. The network exhibited an AUC of 0.97 (95% CI 0.97–0.98) for identifying images with NSAID mucosal ulcers. This is comparable to the previous results for CD patients, with AUCs of 0.94–0.99.

**Figure 5:**
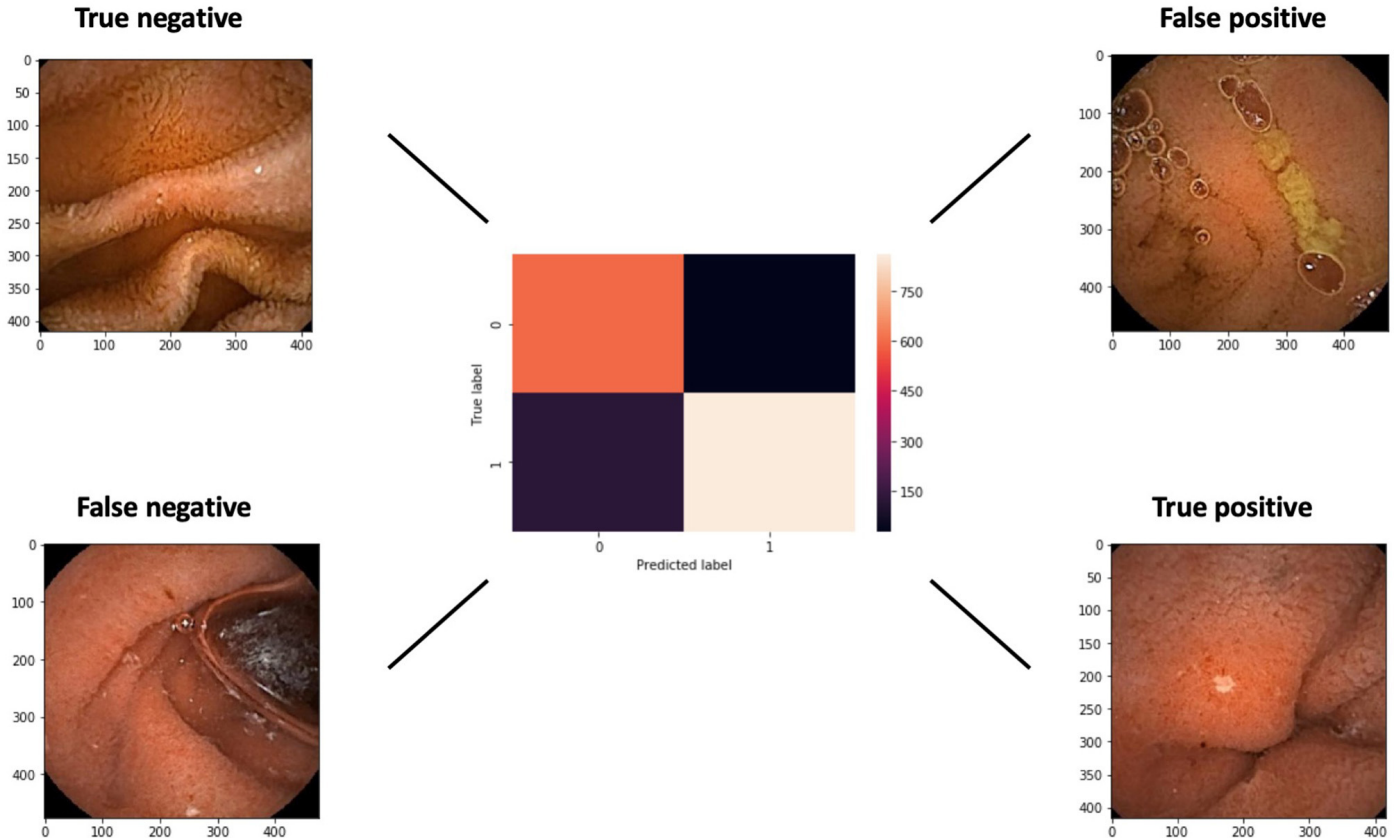
A confusion matrix of the networks results for a cut-off probability of 0.5. Examples of false and true negative and positive images are shown.

A confusion matrix with examples of true and false, negative and positive, images is presented in Figure 5. The false negative example exhibits an ulcer at the edge of the image.

For Youden's index, the network showed sensitivity 92% and specificity 95%. For all specificities cut-off values, a high F1 score of >0.90 was shown (Table 1).

**Table 1:** Metrics of the deep learning network for detecting NSAID-enteropathy.

**Fixed specificity**	**Sensitivity**	**Specificity**	**PPV**	**NPV**	**F1**
Youden's index	0.92 (95% CI: 0.90–0.94)	0.95 (95% CI: 0.94–0.97)	0.97 (95% CI: 0.95–0.98)	0.89 (95% CI: 0.86–0.91)	0.94 (95% CI: 0.93–0.95)
90%	0.94 (95% CI: 0.92–0.95)	0.90	0.93 (95% CI: 0.92–0.95)	0.90 (95% CI: 0.88–0.92)	0.94 (95% CI: 0.92–0.95)
95%	0.92 (95% CI: 0.91–0.94)	0.95	0.97 (95% CI: 0.95–0.98)	0.89 (95% CI: 0.86–0.91)	0.94 (95% CI: 0.93–0.95)
99%	0.85 (95% CI: 0.82–0.87)	0.99	0.99 (95% CI: 0.98–1.00)	0.80 (95% CI: 0.78–0.83)	0.91 (95% CI: 0.90–0.93)

*Metrics (Sensitivity, Specificity, PPV, NPV, F1 score) of the deep learning network probabilities for detecting NSAID-enteropathy in video capsule endoscopy (VCE) images. The different metrics were computed for Youden's index and for fixed specificities of 90, 95, and 99%*.

## Discussion

In the past few years, there has been a surge of publications related to the use of artificial intelligence in gastroenterology and gastrointestinal endoscopy. Several studies have demonstrated the impressive results of detecting ulcers in VCE (16). In recent studies we have found that AI can accurately identify CD ulcers and also rank their severity (17, 18). However, small bowel ulcers are not specific to Crohn's disease, and may be caused by various other etiologies.

NSAID induced enteropathy is a common complication, potentially afflicting >50% of chronic NSAID users and may be detected even in occasional and low-dose NSAID users. NSAID-induced small bowel ulcers are a common cause of occult small bowel bleeding. As NSAID-induced ulcers are morphologically similar to those of CD, this entity should be considered as a potential differential diagnosis for suspected CD.

In order to train a neural network, a large number of images are needed (14). The construction of NSAID ulcers dataset is a complex endeavor. It is also methodologically challenging to generate a ‘clean' dataset of NSAID-induced lesions as other mimicking etiologies are often hard to exclude in real-practice patients. The present study utilized a unique dataset from a clinical trial in which healthy individuals received NSAID and underwent VCE, thereby overcoming the aforementioned methodological constraints. Using healthy individuals, who deliberately ingested aspirin to induce enteric ulcerations as part of a clinical trial is a strong methodological advantage of the present study allowing validated etio-morpholgical comparison with CD ulcers. This stands in contrast with any such future attempt using clinically accrued patients, in whom ascribing ulcers to aspirin or NSAIDs etiology cannot be definitively done in most cases.

The DL network, trained using a large dataset of CD ulcers, achieved an AUC of 0.97 for detecting NSAID-induced ulcers. This prediction capability is similar to the AUC (0.94–0.99) of the network for detecting CD ulcers (17). Our findings corroborate the morphological similarity of CD and NSAID-induced ulcers. Moreover, the network was very fast and efficient in detection of the ulcers, arguably much faster than the expected reading time of a human reader.

Our neural network has proved to be efficient in identifying NSAID induced ulcers; however, it is important to take into account that this system is trained solely on CD ulcers and thereby will identify both types of ulcers as the same entity. The current algorithm will be able to identify ulcers in general and will not be able to specifically determine whether it is a NSAID or a CD induced ulcer. Recently, DL has been implemented in the clinic for GI applications (22). When using this tool, physicians should be aware of the interactions between various pathologies as well as the limitations of AI.

This study has several limitations. This is a retrospective analysis of a prospectively collected data. Prospective interventional studies are needed to evaluate the retrospective results to show real-world usefulness. The study includes a limited number of images, particularly for NSAID induced ulcers. Moreover, DL is a rapidly emerging field, and new algorithms are continuously developed. In this work we've used the Xception model since it was used in a previous related work (17). Yet, this model has several limitations: 1. Xception CNN model network is compatible only with the TensorFlow backend, 2. Xception require 299 × 299 pixel inputs, 3. Xception image weights are between 90 and 100MB. Other models may show better results. Lastly, future projects should prospectively investigate networks in real-life scenarios.

The described method presents a classification model for still VCE images. Future studies should expand this method for entire video analysis to provide a wholistic prediction and to replicate clinical scores such as the Lewis or the CDEIS scores. Moreover, clinical data could be integrated into the final layers of the neural network to augment the visual data.

## Conclusion

A network trained on VCE CD ulcers similarly identified NSAID enteropathy findings. As DL is transforming gastrointestinal endoscopy, this result should be taken into consideration in the future design and analysis of VCE deep learning applications.

## Data Availability Statement

The raw data supporting the conclusions of this article will be made available by the authors, without undue reservation.

## Ethics Statement

The studies involving human participants were reviewed and approved by Cork University. The patients/participants provided their written informed consent to participate in this study.

## Author Contributions

EK had full access to all of the data in the study and takes responsibility for the integrity of the data and the accuracy of the data analysis. UK, EK, and SB-H: concept and design. EK, UK, BM, AD, SS, YB, EK, AG, RY, MB, FS, RE, and SB-H: acquisition, analysis or interpretation of data, critical revision of the manuscript for important intellectual content, and administrative, technical, or material support. UK, EK, and SS: drafting of the manuscript. EK: algorithm design and statistical analysis. UK, EK, SB-H, and RE: supervision. All authors contributed to the article and approved the submitted version.

## Conflict of Interest

UK: speaker and advisory fees- ABBVIE, Jannsen, MSD, Takeda, Medtronic. Research support- Janssen Takeda Medtronic. SB-H: speaker and advisory fees for Takeda, Abbvie, Janssen, Celltrion, GSK, Pfizer and research support from Takeda, Celltrion, Janssen, and Abbvie. SB-H: received advisory and/or research support from Abbvie, MSD, Janssen, Celltrion, Takeda, GSK, Pfizer. RE: received advisory and/or research support from Abbvie, Janssen, Takeda and Medtronic. FS: co-founder of Alimentary Health Ltd, 4D pharma Cork and Atlantia Food Clinical Trials and has been scientific adviser to Kaleido Biosciences Ltd. The remaining authors declare that the research was conducted in the absence of any commercial or financial relationships that could be construed as a potential conflict of interest.

## Publisher's Note

All claims expressed in this article are solely those of the authors and do not necessarily represent those of their affiliated organizations, or those of the publisher, the editors and the reviewers. Any product that may be evaluated in this article, or claim that may be made by its manufacturer, is not guaranteed or endorsed by the publisher.
